# GSA’S CONGRESSIONAL UPDATE

**DOI:** 10.1093/geroni/igac059.813

**Published:** 2022-12-20

**Authors:** Brian Lindberg

## Abstract

This popular annual session will provide cutting-edge information on what the 117th Congress has and has not accomplished to date, and what may be left for the lame duck session to address. Speakers will discuss key issues such as Social Security, Medicare, Medicaid, Older Americans Act, Build Back Better Act, social isolation, serious illness care, and funding. Predictions for the 118th Congress may be provided.

